# After harm involving AI: reconstructing human–AI clinical system performance

**DOI:** 10.1177/01410768261479489

**Published:** 2026-08-20

**Authors:** Bryan D Johnston, Scott J Adams, Jay Kalra

**Affiliations:** 1 Department of Pathology and Laboratory Medicine, College of Medicine, University of Saskatchewan, Saskatoon, Saskatchewan, Canada; 2 Department of Medical Imaging, College of Medicine, University of Saskatchewan, Saskatoon, Saskatchewan, Canada; 3 Royal University Hospital, Saskatchewan Health Authority, Saskatoon, Saskatchewan, Canada

Editor,

We read with great interest Abbasi’s reflection^1^ on responsibility when AI’s ‘coded hand’ is implicated in patient harm, and Macrae’s systems perspective on the same concern.^2^

Macrae argues that explaining how and why AI contributes to harm requires examining the complex sociotechnical systems in which it is embedded, rather than the technology alone. Such analysis must integrate clinical, experiential, management, organisational, technical and regulatory evidence, while considering AI technologies and healthcare professionals in combination as a distributed cognitive system.

The revised London Protocol offers a method adaptable to this task.^3^ It treats an incident or patient journey as a ‘window on the system’, shifting attention from individual actions towards the interplay among human, technical and organisational contributory factors.

We suggest that, after harm involving AI, the patient journey should remain the window on the system, while human–AI clinical system performance becomes the focus of reconstruction. By this, we mean how the AI technology, healthcare professionals and the clinical environment came together as care unfolded.

The challenge is to integrate human and technological evidence so that incident analysis can reconstruct how the human–AI clinical system performed within the circumstances at the time. The aim is to identify the system as experienced and establish how its output entered clinical decision-making.

Incident analysis does not ordinarily require complete access to a clinician’s cognitive process; it reconstructs decisions and actions from available evidence. Similarly, understanding harm involving AI need not require examining every internal computation of the technology, but sufficient evidence to explain how and why it happened. Clinical AI may therefore require new obligations for logging, retention and access to evidence, but not a separate response to harm. Before responsibility can be assigned, the patient journey should guide reconstruction of the human–AI clinical system as it operated when the harm occurred.

## Declarations

**Provenance:** Not commissioned; editorial review.
